# Improved Stability and Manufacturability of Nucleocapsid Antigens for SARS-CoV2 Diagnostics through Protein Engineering

**DOI:** 10.3390/biom13101524

**Published:** 2023-10-14

**Authors:** Esha Shukla, Lipsa Choudhury, Saurabh Rastogi, Arshmeet Chawla, Sanghati Bhattacharya, Umesh Kaushik, Manan Mittal, Anurag Singh Rathore, Gaurav Pandey

**Affiliations:** 1 Bioprocess and Bioproduct Development Laboratory, University School of Biotechnology, Guru Gobind Singh Indraprastha University, Dwarka, New Delhi 110078, India; eshashukla2609@gmail.com (E.S.); lipsachoudhury@gmail.com (L.C.); rastos4@rpi.edu (S.R.); achawla@uchc.edu (A.C.); 2 Department of Chemical Engineering, Indian Institute of Technology New Delhi, Hauz Khas, New Delhi 110016, India; 3 Medsource Ozone Biomedicals Pvt. Ltd., Parmeshwari Colony, Faridabad 121003, India; rnd@ozonebio.com (U.K.); manan@ozonebio.com (M.M.)

**Keywords:** SARS-CoV2, nucleocapsid protein, stability, Lateral Flow Immunoassay, point-of-care, Immune Epitope Database, *E. coli*

## Abstract

The COVID-19 pandemic has had a significant impact on human health management. A rapid diagnosis of SARS-CoV2 at the point-of-care (POC) is critical to prevent disease spread. As a POC device for remote settings, a LFIA should not require cold-chain maintenance and should be kept at normal temperatures. Antigen stability can be enhanced by addressing instability issues when dealing with fragile components, such as proteinaceous capture antigens. This study used immunologically guided protein engineering to enhance the capture nucleocapsid (NP) antigen stability of SARS-CoV2. A search of the IEDB database revealed that antibodies detecting epitopes are almost uniformly distributed over NP_1-419_. In contrast, N-terminal stretches of NP_1-419_ are theoretically more unstable than C-terminal stretches. We identified NP_250-365_ as a NP stretch with a low instability index and B-cell epitopes. Apart from NP_1-419_, two other variants (NP_121-419_ and NP_250-365_) were cloned, expressed, and purified. The degradation pattern of the proteins was observed on SDS-PAGE after three days of stability studies at −20 °C, 4 °C, and 37 °C. NP_1-419_ was the most degraded while NP_250-365_ exhibited the least degradation. Also, NP_1-419_, NP_250-365_, and NP_121-419_ reacted with purified antibodies from COVID-19 patient serum. Our results suggest that NP_250-365_ may be used as a stable capture antigen in LFIA devices to detect COVID-19.

## 1. Introduction

The COVID-19 pandemic had a global impact on not only human health but the economics of countries and everyday livelihoods as well. Disease surveillance is one of the key tools used to manage pandemics and is an integral part of national health management systems worldwide. Having robust stability regarding point-of-care (POC) diagnostics in remote settings is the key to effective surveillance. This helps to curb the spread of pandemics and we have seen its importance in the largest pandemic of SARS-CoV2 (COVID-19) [1]. To stop the spread of the disease, it was necessary to detect the disease in patients, quarantine, and prioritize the vaccine implementation program. As a fast and easy-to-use diagnostic tool, the Lateral Flow Immunoassay (LFIA) has caught the attention of many scientists. It has an advantage over other POC techniques as it covers the WHO ASSURED criteria of POC testing in resource-limited settings [2]. A LFIA, an analytical tool for the onsite detection of target substances, has advantages including rapidity, simplicity, and relative cost-effectiveness [3].

The coronavirus disease of 2019 (COVID-19) is caused by novel coronavirus SARS-CoV2, a beta-coronavirus with ssRNA genomes that encode sixteen different non-structural proteins (NSPs) and four essential proteins named spike glycoprotein (S), small envelope protein (E), matrix protein (M), and nucleocapsid protein (NP or N-protein) [1,4]. A number of diagnostic tests were developed based on molecular diagnostics, antigens, and serology in response to the COVID-19 pandemic to detect the SARS-CoV2′s virus, antigens, or antibodies generated in response to infection. Studies suggest that the majority of patients develop an antibody response only in the second week after the onset of symptoms [5]. A diagnosis of the COVID-19 infection based on antibody response is often only possible in the recovery phase. This is very useful for antibody detection to establish their usefulness in disease surveillance and epidemiologic research. Vaccines that have proven to be immunogenic incorporate spike protein as the functional antigen over nucleocapsid. To distinguish the antibody response from natural infection and vaccine immunization, the nucleocapsid protein must be used as a diagnostic antigen. Nucleocapsid is used for serology tests due to its abundant expression and conservation in the genome. The high titer antibodies against the N-protein are found in SARS-CoV2 infected patients, which can be detected as a biomarker for infection through various diagnostic tools; these N-specific antibodies dominate the overall antibody response [6,7].

The full-length nucleocapsid protein was expressed and purified from bacterial cells in both forms, soluble and as inclusion bodies (IBs). To check the reactivity of both forms of protein to COVID-positive patient serum, an ELISA was developed and the IB form was found to be slightly more reactive [8]. Researchers have purified the whole nucleocapsid and N-terminal domain for serological assay development. Biochemical studies, such as static light scattering, small-angle X-ray scattering (SAXS) and size exclusion chromatography, confirm that the N-protein is largely present in its dimeric form [9]. The refolded N-protein (IB form) was also used for the preliminary detection of infection [1].

According to published studies, the first 40 amino acids of the N-protein sequence are intrinsically disordered. The recombinant fragmented NP (rfNP) devoid of disordered regions (1–40 aa) was expressed and purified in *E. coli* in the soluble form. ELISA assay validation with 68 negative and 50 positive patient samples was performed. This showed that rfNP is also useful for the detection of SARS-CoV2 antibodies during infection [10]. Studies also proved that the N-terminally truncated nucleocapsid protein is a better serological marker than the complete N-protein in evaluating SARS-CoV2 immunogenicity [11].

One of the studies also elucidated the impact of the dimerization of SARS-CoV2 nucleocapsid protein on the sensitivity of ELISA-based COVID-19 diagnostics and showed that the dimeric form of the N-protein shows higher stability and antigenicity than the monomeric form [12].

Although efforts have been made to understand infection and immunity mechanisms based on N-protein peptide structure, little has been done to integrate these insights into the development and manufacturing of a stable N-protein to be used in remote settings as a capture antigen for diagnostics. A full-length recombinant N-protein is expressed as inclusion bodies in *E. coli*. Incubation temperature and medium composition were optimized for soluble expression. However, the purified protein remained unstable due to aggregation and proteolytic degradation (lab data in Supplementary File). Unstable capture antigens may not be useful for diagnostic use as they may not have consistency in outcome and may compromise the specificity and sensitivity of the assay.

In our study, to identify the stable and immune-reactive version of the N-protein, we first found computationally predicted linear B-cell epitopes based on the antigen sequence characteristics of the N-protein from the Immune Epitope Database (IEDB) Analysis Resource website. Then, we integrated the peptide instability index information to predict a stable region of N-protein comprising linear B-cell epitopes. For each variant involved, we also integrated the information about variants from UniProt. Based on this analysis, we identified two probable variants of the full-length nucleocapsid protein representing two different regions of the nucleocapsid protein. Using *E. coli*, these two variants, along with the native full-length N-protein, were successfully expressed and purified. The stability of all three purified proteins was then evaluated in wet lab experiments. Among the three stretches of the N-protein, the smallest region with the least epitope binding sites (according to IEDB analysis) was found to be stable as compared to other constructs at different temperature conditions.

## 2. Materials and Methods

### 2.1. Computational Analysis and Websites

#### 2.1.1. UniProt and Protter

The protein sequence of SARS-CoV2 nucleocapsid was obtained from the UniProt database (https://www.uniprot.org/ initially accessed on 30 April 2020 and rechecked on 6 August 2023) and the variant data retrieved for the SARS-CoV2 nucleocapsid protein were used for further analysis. The variants of nucleocapsid protein are visualized using the online tool Protter (https://wlab.ethz.ch/protter/# last accessed on 6 August 2023) [13].

#### 2.1.2. The Immune Epitope Database (IEDB)

The Immune Epitope Database (IEDB) is the online computational software (https://www.iedb.org/ initially accessed on July 2021 and rechecked on 6 August 2023) used for the prediction of linear B-cell epitopes in the nucleocapsid protein sequence (NP_1-419_) of SARS-CoV2 [14,15]. The BepiPred-2.0 server predicts B-cell linear epitopes from a protein sequence, using a random forest algorithm trained on epitopes and non-epitope amino acids determined from crystal structures. Sequential prediction smoothing is performed afterwards. The residues with scores above the threshold (default value is 0.5) are predicted to be part of an epitope. The Ê values of the scores are not affected by the selected threshold.

#### 2.1.3. Instability Index and Isoelectric Point

Instability Index: The instability index provides an estimate of the stability of the protein in a test tube. Previously it has been revealed that there are certain dipeptides, the occurrence of which is significantly different in the unstable proteins compared with those in the stable ones. In this method, a weight value of instability is assigned to each of the 400 different dipeptides (DIWV). Using these weight values, it is possible to compute an instability index (II). A protein whose instability index is smaller than 40 is predicted to be stable; a value above 40 predicts that the protein may be unstable [16,17]. The instability index (II) and isoelectric point were calculated using the online tool ProtParam (https://web.expasy.org/cgi-bin/protparam initially accessed on 30 April 2020 and rechecked on 6 August 2023).

### 2.2. Strains, Plasmids, Chemicals, and Filters

*E. coli* DH5α [F+ endA1 glnV44 thi-1 recA1 relA1 gyrA96 deoR nupG purB20 φ80dlacZΔM15 Δ(lacZYA-argF)U169, hsdR17(rK–mK+), λ] was used for sub-cloning and plasmid construction. BL21(DE3) [F– ompT gal dcm lon hsdSB(rB–mB–) λ(DE3 [lacI lacUV5-T7p07 ind1 sam7 nin5]) [malB+]K-12(λS)] was used for the expression of the fusion protein. Plasmid pET-28b with 6Xhis tag was used for the expression of the fusion proteins. The SARS-CoV2 nucleocapsid protein gene was cloned in the restriction sites *NcoI* and *BamHI* in the pET-28b vector, which was synthesized from GenScript. Details of the hosts and plasmids are given in Table 1. Restriction endonucleases, Agarose ITM (cat No.: 0710), acrylamide (cat No.: 0341), bis-acrylamide (cat No.: 0172), sodium dodecyl sulfate (SDS) (cat No.: 0227), ammonium per sulfate (APS) (cat No.: 0486), isopropyl β-D-1-thiogalactopyranoside (IPTG) (cat No.: 0487), and TEMED (cat No.: 0761) were purchased from Amresco, Framingham, MA, USA. The GeneJET plasmid miniprep kit (cat No.: K0502) and GeneJET gel extraction kit (cat No.: K0691) were purchased from Thermo Fisher Scientific, Lithuania, Europe. Luria Bertani (LB) medium (cat No.: M1245) and kanamycin (cat No.: MB105) were purchased from HiMedia, Mumbai, India. Sodium hydroxide (cat No.: 68451) and ethylene diamine tetra-acetic acid (EDTA) (cat No.: 054960) were purchased from SRL, Gurugram, India. All other chemicals and buffer salts were procured from Merck-Millipore, Darmstadt, Germany. Amicon^®^ ULTRAcel^®^ 10K (cat No.: UFC901096) and 3K (cat No.: UFC900324) centrifugal filters were purchased from Merck-Millipore, Cork, Ireland and restriction enzymes, ligase, and polymerase enzymes were purchased from Fermentas, Vilnius, Lithuania. The PierceTM BCA protein assay kit (cat No.: 23227) was procured from Thermo Fisher Scientific, Tokyo, Japan and NuviaTM IMAC Ni-Charged Resin (cat No.: 780-0800) was procured from Bio-Rad, Feldkirchen, Germany.

### 2.3. Construction of Fusion Nucleocapsid Protein (NP_1-419_) and its Variants (NP_121-419_ and NP_250-365_)

The SARS-CoV2 nucleocapsid gene NP_1-419_ (codon-optimised for *E. coli*) was procured from GenScript, Piscataway, NJ, USA. The cloning of its two variants, NP_121-419_ and NP_250-365_, was completed in-house in the pET-28b vector with the 6Xhis tag at the C-terminal. The NP_121-419_ and NP_250-365_ were amplified from the nucleocapsid gene plasmid with their respective primers. The primers’ details are provided in Table 2. The PCR amplification for NP_121-419_ consisted of 30 cycles, where each cycle consisted of a denaturation step at 94 °C for 30 s, annealing at 62 °C for 30 s, and elongation at 72 °C for 45 s. This was followed by a final extension step at 72 °C for 5 min. Amplified genes and vectors were digested with restriction enzymes and ligated suitably. The digested vector and the insert were purified with the gel extraction process and ligated in the presence of the T4 DNA ligase enzyme. The ligated product was transformed in *E. coli* DH5α cells for the screening of the positive recombinant clones. Positive clones, pET-28-NP_121-419_ and pET-28-NP_250-365_, were confirmed with colony PCR with similar PCR conditions to those mentioned above. All of the positive recombinant clones were confirmed through Sanger sequence analysis. The glycerol stocks for positive clones were prepared and stored at −70 °C or below for further use.

### 2.4. Expression Study of Nucleocapsid Protein and Its Variants (NP_1-419_, NP_121-419_ and NP_250-365_)

The positive clones of pET-28-NP_1-419_ and the variants pET-28-NP_121-419_ and pET-28-NP_250-365_ were transformed in BL21(DE3) competent cells. The transformed colonies were grown overnight in Luria broth medium at 25 °C and 200 rpm. The overnight cultures were inoculated in secondary media, which was Terrific broth (TB) 1000 mL with 1% glycerol at 25 °C and 200 rpm. Cultures were allowed to grow until the OD at 600 nm (OD_600_) reached 1, followed by the addition of 3% ethanol in the culture. Again, the cells were allowed to grow until the OD_600_ reached 1.5 and was induced with 0.5 mM IPTG. Additionally, 16 h post-induction, the samples were collected for the evaluation of protein expression. According to the final OD of the cells, 10^8^ cells/mL were collected from each flask, centrifuged at 8000× *g*, and resuspended in 100 µL of reduced SDS-loading buffer. The samples were boiled for 10 min, centrifuged at 8000× *g*, and the supernatant was loaded on the 12% SDS-PAGE to check the expression of the cells. The rest culture was harvested by centrifuging at 8000× *g* for 30 min at 4 °C. The lysis buffer (10 mM sodium phosphate pH 8, 150 mM NaCl, 0.25% Triton X100, 5 mM benzamidine, and 0.2% lysozyme) was added and sonicated in an ice bath. The soluble fraction of the protein was collected after centrifugation at 10,000× *g* for 30 min at 4 °C for further purification with IMAC.

### 2.5. Purification of Nucleocapsid Protein and Its Variants (NP_1-419_, NP_121-419_ and NP_250-365_) with Immobilized Metal Affinity Chromatography (IMAC)

The soluble fractions of NP_1-419_, NP_121-419_, and NP_250-365_ were filtered with 0.45 µ filters. The filtered fraction was loaded on the FPLC column (ÄKTA start, Cytiva, Maralborough, USA) packed with Ni-NTA resin (Biorad, California, USA) pre-equilibrated with five column volumes (CVs) of 10 mM sodium phosphate buffer pH 8, 50 mM NaCl, 5 mM benzamidine, and 5 mM imidazole at a flow rate of 0.5 mL/min. Flow through was collected separately to further analyze the unbounded proteins. The column was washed with 5 CV base buffer containing 10 mM sodium phosphate, 5 mM benzamidine, 50 mM NaCl, and 5 mM imidazole. Additionally, 2 CV elution at 0.5 mL/min was completed at an imidazole gradient of 20–500 mM. The eluted fractions were collected and analyzed on 12% SDS-PAGE [18,19]. The samples having the band of protein with a corresponding size to that of NP_1-419_, NP_121-419_, and NP_250-365_ were pooled down, respectively, and buffer was exchanged with 10 mM sodium phosphate buffer pH 8 and 150 mM NaCl buffer.

### 2.6. Qualitative and Quantitative Estimations of the Purified Proteins

The quantitative estimation of the purified protein was conducted with the Pierce BCA kit (Thermo Fisher Scientific) according to the method mentioned by the kit manufacturer. The purified NP_1-419_, NP_121-419_, and NP_250-365_ were analyzed by SDS-PAGE, using a 12% SDS-polyacrylamide gel under a reducing (25% β-mercaptoethanol, *v*/*v*) condition for the qualitative estimation of the purified protein. The molecular weights of the proteins were estimated with suitable markers. The gel was stained with Coomassie blue R-250.

### 2.7. Activity of the Purified Nucleocapsid Protein and Its Variants with the COVID-19 Positive Sera

(i) Enzyme-linked Immunoassay (ELISA): The proteins generated in this study, along with the positive control protein (full-length nucleocapsid protein obtained from Fapon Biotech, China) and negative control protein (glutathione S-transferase), were further analyzed for their activity with COVID-positive serum. In addition, 50 µL of each protein (stock 2 µg/mL) was coated in duplicate on the high binding ELISA plate with carbonate buffer pH = 9.5 and incubated overnight at 4 °C. The next day, the plate was washed with 100 µL of wash buffer (1XPBS 0.1% Tween 20). Then, 200 µL of blocking buffer (3% skimmed milk in 1XPBS) was added to the plate and incubated at RT for 1 h. After the blocking step, the plate was washed thrice with the wash buffer. Following the washing step, 50 µL of COVID-positive sera (1:50 dilution) was added to each well and the plate was further incubated at RT for 1 h. After primary antibody incubation, the plate was washed 6 times with the wash buffer. Then, 100 µL of secondary antibody anti-human antibody Sigma A0170 (1:10,000) dilution was added to each well. The plate was incubated for 1 h at RT. After incubation, the plate was washed 6 times with wash buffer. Then, 100 µL of TMB/H_2_O_2_ was added to each well and incubated at RT for 10 min. Following this, 100 µL of stop solution (1 M sulphuric acid) was added to each well and the absorbance was measured at 450 nm with reference to 650 nm.

(ii) Lateral Flow Immunoassay (LFIA): LFIAs were prepared to check the sero activity of recombinant proteins with COVID-positive sera. Here, a strip containing an absorbent pad, nitrocellulose membrane, conjugate pad, and sample pad was placed inside the plastic housing/cassette. The nitrocellulose membrane was immobilized with goat anti-rabbit IgG at control line position ‘C’ and anti-human IgG at test line position ‘2′. A gold conjugate pad containing colloidal gold sol conjugated with recombinant N-protein and Rabbit IgG was prepared. Test strips of 3.0 mm were prepared and housed in a plastic cassette.

Colloidal gold sol was prepared using gold chloride and sodium citrate. The pH of the colloidal gold solution was adjusted to pH 8.0 ± 0.2 using potassium carbonate. Recombinant protein at the concentration of 4.0 mg protein was added slowly into 1000 mL of pH-adjusted colloidal gold sol kept in a beaker on a magnetic stirrer. The colloidal gold sol containing recombinant protein was allowed to mix under mild stirring conditions for one hour. A scan on a spectrophotometer was conducted to check the maxima absorbance peak at 530 ± 5 nm. Unbound sites of the colloidal gold were blocked with the addition of BSA (bovine serum albumin) at a final concentration of 2% and the entire solution was kept on gentle mixing for half an hour. The entire solution was centrifuged. After centrifugation, the supernatant was discarded and pellets were reconstituted in the 10 mM Tris buffer with a pH of 8.0 containing BSA, Tween-20, sucrose, and trehalose. A cocktail of two conjugates (12 OD recombinant nucleocapsid proteins and 3 OD of Rabbit IgG) was prepared for drying on a conjugate pad with a 5 mm width. The strip of the conjugate pad was impregnated with the cocktail of colloidal gold conjugates and dried under a humidity control area for further use along with a nitrocellulose membrane immobilized with goat anti-rabbit IgG at control line region C, anti-human IgG at test line region ‘2′, and anti-human IgM at test line region ’1′.

Tests were run using known COVID-positive and -negative serums obtained from SS serum, India. A 10 µL sample was added the to sample well (S) of the plastic cassette/device followed by 1–2 drops of assay buffer (50 mM Tris buffer, pH 8.5 containing biological detergents and preservatives). The results were read within 15–20.

### 2.8. Protein Stability Studies

The purified proteins were also checked for their stability at different temperatures for 3 days. In addition, 10 µg of each protein was stored in 3 different sealed tubes at different temperatures (−20 °C, 4 °C, and 37 °C) for 3 consecutive days. The temperature of each set was constant. Once the incubation period was over, samples were finally analyzed on 12% SDS-PAGE for their stability as well as degradation at different temperatures.

The protein samples charged under stability were also analyzed for mass using an Agilent 1260 Infinity Bio-inert Quaternary LC system made up of a quaternary pump with a degasser, an autosampler with a cooling unit, and a diode array detector (DAD) connected to an Agilent 6230 ESI-TOF-MS instrument, RP-HPLC; the intact mass was measured with an AdvanceBio RP-mAb C8 column. The column was saturated with 90% mobile phase A (0.1% *v*/*v* formic acid (FA) in MilliQ) and 10% mobile phase B (0.1% *v*/*v* FA in acetonitrile) prior to injection. Samples were filtered through 3 kDa Nanosep^®^ centrifugal filters and the buffer was swapped to 0.1% *v*/*v* FA in MilliQ (Pall Corporation, New York, USA). The sample was placed onto the column at a concentration of 1 mg/mL and it was separated using a linear gradient of 10 to 60% B over 35 min at a flow rate of 0.5 mL/min. Monitoring UV absorbance at 280 nm and recording TIC for 1000–7000 m/z were used to achieve the detection. Prior to analysis, positive ion mode calibration for MS spectra was performed. The voltage for the fragmentor (Vfrag) was set to 400 V and the capillary gas voltage (Vcap) was set to 350 °C at 5500 V. The MS spectra were deconvoluted using the Agilent MassHunter Qualitative Analysis and BioConfirm software, ver 10.0, utilizing maximum entropy (MaxEnt) and peak modelling (pMod) algorithms.

## 3. Results and Discussion

### 3.1. Computational Analysis and Identification of the Stable Region of the Nucleocapsid Sequence

**Figure 1:**
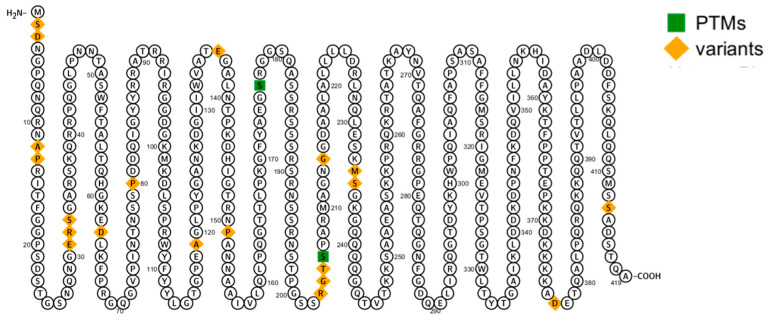
Schematic diagram of nucleocapsid protein sequence generated by Protter.

Under Identity P0DTC9, a sequence of 419 amino acids was retrieved for the SARS-CoV2 nucleocapsid protein from the UniProt database. Figure 1 shows a nucleocapsid protein with variants and Post translational modifications (PTMs). Twenty amino acids of the nucleocapsid are different in the variants, along with two additional PTM positions (176,206) (Figure 1).

**Figure 2:**
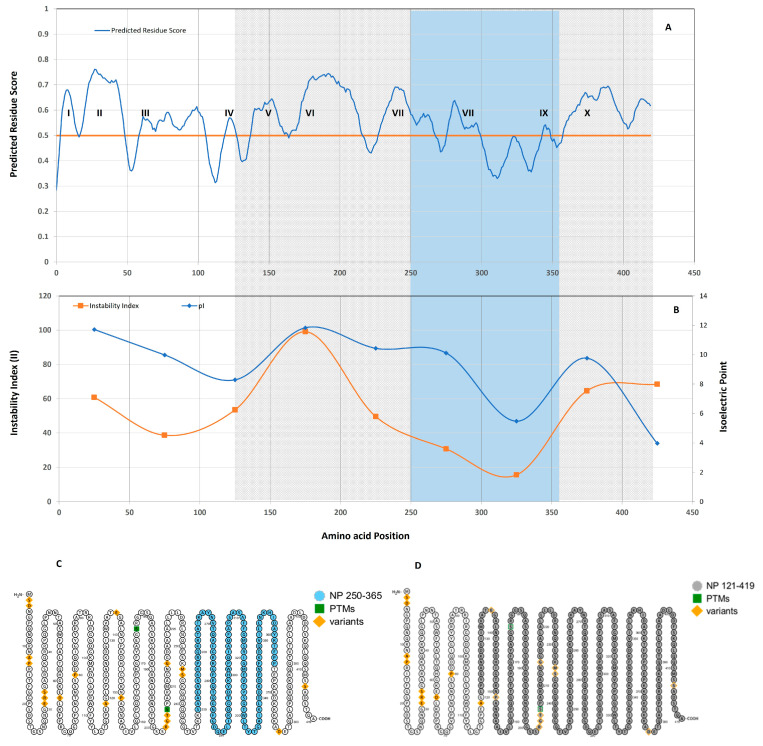
(**A**) Based on the IEDB analysis tool, the 10 most probable epitope regions numbered as I–X were identified for the complete nucleocapsid protein (NP_1-419_), orange line depicts the threshold value of predicted score above which there is the occurrence of the B-cell epitope; (**B**) shows the instability index and the isoelectric point distribution over the complete nucleocapsid protein; each observation point represents the outcome of 50 amino acid stretches. The blue shaded region shows an instability index of less than 40%, depicting a stable protein region and corresponding to amino acids from positions 250–365. This subsequently points to three regions (VII, VIII, and IX) with B-cell epitopes. The grey shaded region shows the amino acids positions from 121–419 and does not contain the N-terminal region of nucleocapsid. Schematic figure of the nucleocapsid protein generated by Protter showing (**C**) 299 amino acids of NP_121-419_ protein in blue and (**D**) 155 amino acids of NP_250-365_ protein in grey.

IEDB analysis shows linear epitopes almost uniformly distributed across a nucleocapsid polypeptide stretch. Out of 419 amino acids, the predicted score of 312 amino acids is above 0.5, as calculated by the Bepipred Linear Epitope Prediction 2.0 algorithm of the IEDB database. Therefore, it theoretically may contribute to the linear epitopes of nucleocapsid proteins. There are 10 regions (I to X) on the nucleocapsid, which can contribute to epitope formation as shown (Figure 2A). Further, the instability index, as well as the isoelectric point of the nucleocapsid polypeptide, was mapped. For mapping, the stretches of 50 amino acids over the whole polypeptide were used to calculate the instability index (II) and pI. To calculate, starting from the first amino acid of the nucleocapsid protein, a stretch of 50 amino acids from Position 1 to Position 50 was chosen for calculation, followed by another stretch of 50 amino acids starting after the 25th position, from Position 26 to Position 75, and continuing until the complete polypeptide length was spanned. The data obtained are shown in Figure 2B. In Figure 2B, the instability index drops below 40 (characteristics of stability) between 250 and 350 amino acids, only in one instance in a complete polypeptide, corresponding to the amino acid positions illustrated in Figure 2A. It corresponds to Regions 7, 8, and 9 out of a total of 10 regions available. Based on this information, it was decided upon to make a truncated protein comprising of NP_250-365_ (Figure 2C). Since the designed protein is comprised of just three regions where the predicted residue score is above 0.5 for the occurrence of the B-cell epitope, it was decided to add another candidate protein for studying. This was achieved by including the surrounding regions with high values regarding predicted residue score. Due to the high variability of the first 120 amino acids in polypeptide, this region was avoided; thus, the NP_121-419_ region was chosen (Figure 2D) (Table 3).

### 3.2. Nucleocapsid Protein Variant Expression Studies

**Figure 3:**
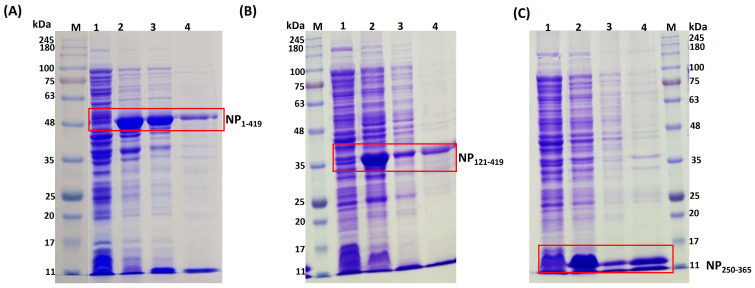
SDS-PAGE with 12% tris glycine for expression analysis. (**A**) NP_1-419_, (**B**) NP_121-419_, (**C**) NP_250-365_. Lane M: molecular marker, Lane 1: 0 h pre-induction, Lane 2: 16 h post-induction, Lane 3: supernatant after lysis, Lane 4: IB fraction obtained after centrifugation of lysate.

To evaluate our hypothesis for the theoretical designs of stable constructs, experiments with actual variants were carried out. *E. coli* BL21(DE3) cells were used to express NP_1-419_, NP_121-419_, and NP_250-365_ proteins. A schematic diagram showing cloning strategies and expression is available in Supplementary Figure S1. In SDS-PAGE gels, NP_1-419_, NP_121-419_, and NP_250-365_ proteins exhibit prominent bands corresponding to 48 kDa, 35 kDa, and 15 kDa, respectively. After sixteen hours of induction, protein partitioning into soluble and inclusion body (IB) fractions was analyzed over SDS-PAGE after the sonication of induced samples (Figure 3). Afterward, the soluble fractions were purified using IMAC chromatography.

### 3.3. Purification of Proteins NP_1-419_, NP_121-419_, and NP_250-365_

**Figure 4:**
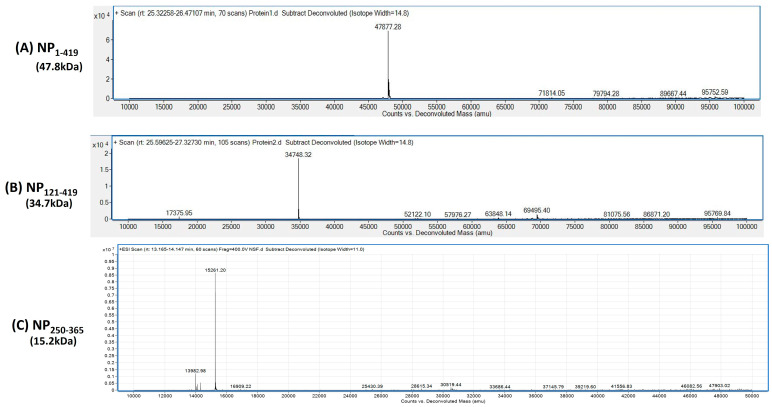
Intact mass analysis of (**A**) NP_1-419_, (**B**) NP_121-419_, and (**C**) NP_250-365_ proteins.

**Figure 5:**
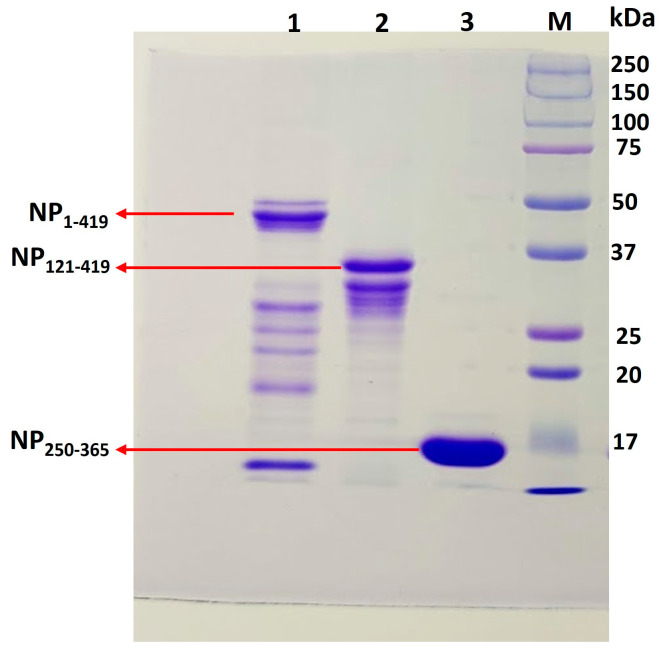
Analysis of purified fusion proteins (a week stored in 10 mM phosphate elution buffer pH 8.0) on 12% SDS-PAGE (each protein is loaded 10 µg) Lane 1- NP_1-419_ protein, Lane 2- NP_121-419_ protein, Lane 3- NP_250-365_ protein, Lane M- molecular marker.

Cells from the induced culture were harvested and resuspended in a lysis buffer. Cell lysates prepared by sonication were clarified by centrifugation before loading onto an IMAC column. The purified soluble proteins eluted from the IMAC columns at different imidazole concentrations were collected separately for further analysis. All eluted samples were analyzed through 12% SDS-PAGE under non-reducing conditions. Chromatograms, along with SDS-PAGE gels, are shown in Supplementary Figures S2A–C. The elution with the protein bands corresponding to its respective size was pooled before the buffer was exchanged with formulation buffer (PBS). For characterization, the intact masses of purified proteins were determined by LC-MS and were found to be 47.8 kDa, 34.7 kDa, and 15.2 kDa for NP_1-419_, NP_121-419_, and NP_250-365_, respectively (Figure 4). Further, the sequence identities were confirmed by peptide mapping and the data are shown in Supplementary Figure S4. At the time of purification, for each of the proteins, no significant degradation was found in the final elution (Supplementary Figures S2A–C). However, for further comparative stability studies of the proteins, purified proteins (NP_1-419_, NP_121-419_, and NP_250-365_) were stored at −20 °C. Some prior degradation was observed, as shown in Figure 5. The same profile was considered as a reference point for the start of stability studies as a function of temperature. However, to investigate the nature of the impurities, we repeated the experiment and performed Western Blots. As these impurities are product-related and react specifically to SARS-CoV2-positive serum, they did not affect seroactivity. Before Western Blots are developed, proteins are transferred to membranes and stained with Ponceau stain. The Ponceau and blots show the same band, indicating that those impurities are primarily product-related (degraded nucleocapsid fragments). The signal obtained for the small protein during Western Blotting was lower than that obtained during the ELISA. We expected this since the probability of accessing the required epitopes in a denatured state in the ELISA and LFIA is higher than in Western Blotting. Refer to the SUP figure in the Supplementary File.

### 3.4. Reactivity of Nucleocapsid Protein and Its Variants against the COVID Sera

**Figure 6:**
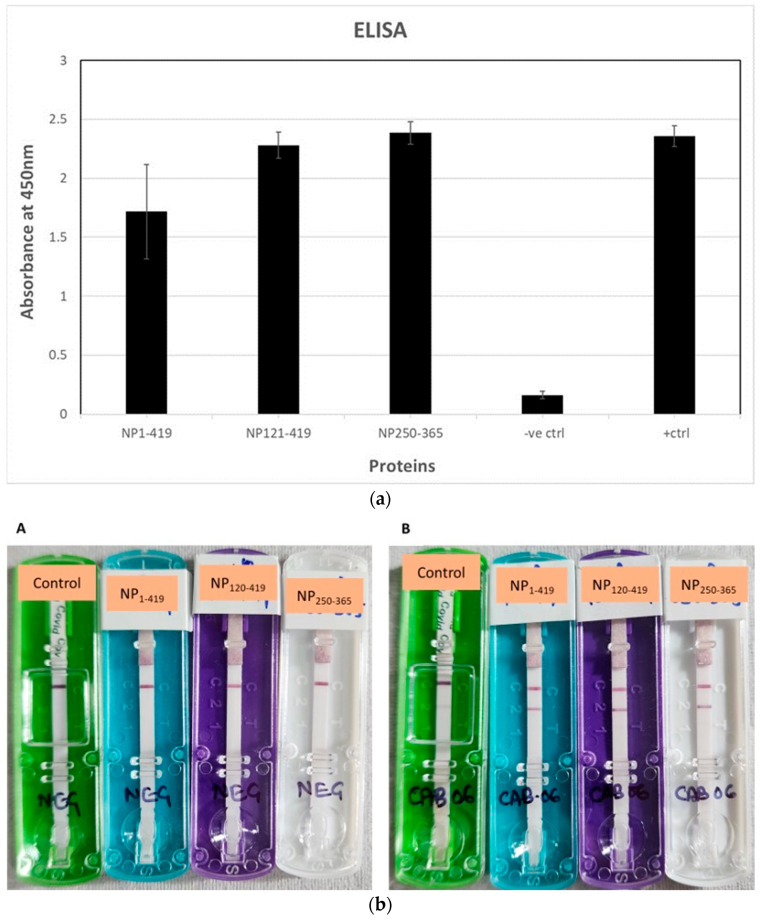
(**a**). Comparative analysis of the ELISA activity of SARS-CoV2 human sera against the three nucleocapsid proteins (NP_1-419_, NP_121-419_, and NP_250-365_) with respect to the positive control protein (FPZ0513) and negative control (GST protein). (**b**). Lateral flow immunochromatographic strips in cassettes showing the comparative analysis of the three nucleocapsid proteins NP_1-419_ (blue cassette), NP_121-419_ (purple cassette), and NP_250-365_ (white cassette) with respect to the control (green cassette, commercialized product in the market). (**A**) Strips tested with SARS-CoV2 human negative sera; (**B**) strips tested with SARS-CoV2 human positive sera.

Both truncated variants and the full-length nucleocapsid protein purified as explained were tested for their reactivity against COVID-positive sera. Our hypothesis was to determine whether the designed variant shows sero reactivity. To check the reactivity, two tests were performed, an Enzyme-linked Immunoassay (ELISA) and Lateral Flow Immunoassay (LFIA). Through the ELISA, it was confirmed that both the truncated variants and the full-length protein were being identified by the SARS-CoV2-positive sera. NP_121-419_ showed slightly higher activity than the other two. The reactivity of NP_1-419_ and NP_250-365_ were almost similar (Figure 6a).

Similar results were obtained when these proteins were tested on the LFIA device. When the SARS-CoV2-positive sera was run on the kit impregnated with the recombinant proteins (NP_1-419_, NP_121-420_, and NP_250-365_) separately along with the negative control, the appearance of the pink/purple line was seen in the test lane with all of the three proteins. These tests have been evaluated with an existing commercially available validated device (Figure 6b). This confirms that all of the three proteins were active against the COVID infection and can be used for the development of diagnostics.

### 3.5. Protein Stability Studies

**Figure 7:**
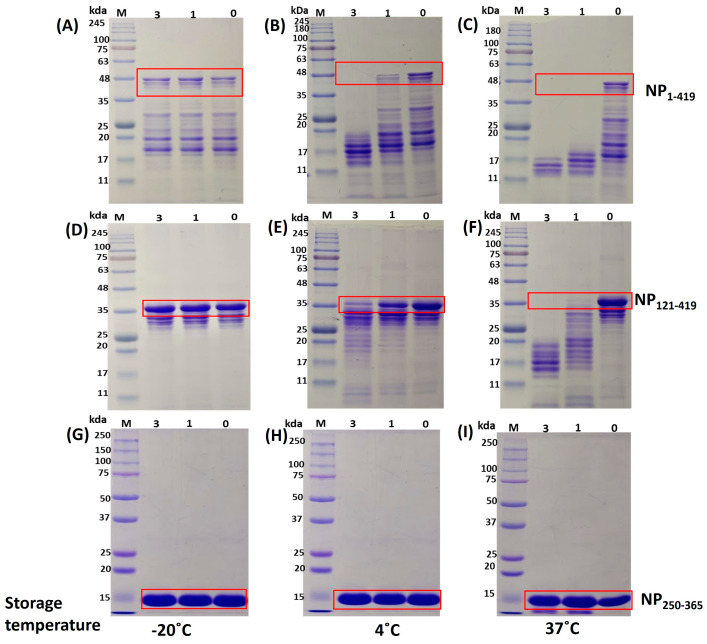
Degradation pattern of NP_1-419_, NP_121-419_, and NP_250-365_ proteins analyzed on 12% SDS-PAGE: in each lane, 10 µg of protein was charged for stability at different temperatures. NP_1-419_ Gel (**A**–**C**): temperatures of −20 °C, 4 °C, and 37 °C, respectively. NP_121-419_ Gel (**D**–**F**): temperatures of −20 °C, 4 °C, and 37 °C, respectively. NP_250-365_ Gel (**G**–**I**): temperatures of −20 °C, 4 °C, and 37 °C, respectively. In each gel, Lane 1–4: molecular marker, Day 3, Day 1, and Day 0 samples, respectively.

All of the three proteins purified above were also compared for stability for three consecutive days at three different temperatures. Around 10 micrograms of protein from each sample were incubated at three different temperatures for three days. The study temperatures were −20 °C, 4 °C, and 37 °C. The SDS-PAGE of 12% tris-glycine revealed different degradation patterns for each protein (Figure 7). We found that degradation was not prominent for NP_1-419_, NP_121-419_, and NP_250-365_ proteins at −20 °C. It may be noted that we had earlier observed some degradation of samples for NP_1-419_ and NP_121-419_ before the start of the experiment. This was during storage at −20 °C for 7–10 days. However, degradation was quite prompt for NP_1-419_ and NP_121-419_ at 4 °C and 37 °C. NP_250-365_ seems to be stable at all temperatures for 3 days. We repeated and included stability studies with SDS-PAGE analysis and mass analysis simultaneously. Degradation signatures observed on SDS PAGE are similar to those observed on mass analysis, with mass better representing the actual mass of fragments. The Supplementary File contains the figures. This suggests that the NP_250-365_ variant may be more suitable because of its stability during manufacturing and storage.

### 3.6. Stabilization of Proteins through Formulation

**Figure 8:**
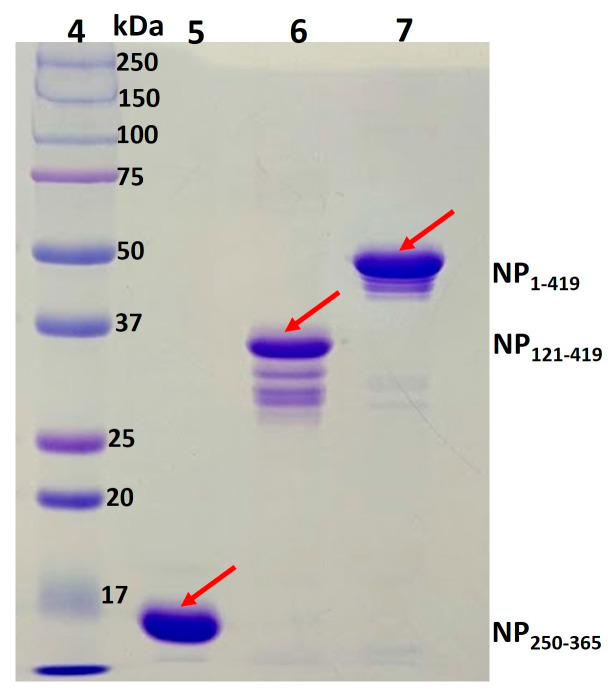
Purified protein stored with different buffer formulations for 1 week at −20 °C on 12% SDS-PAGE., Lane 4: molecular marker; Lanes 5–7: borax buffer formulations of NP_250-365_, NP_121-419_, and NP_1-419_ proteins, respectively. Outcomes of phosphate buffer are shown in Figure 5.

In another approach, NP_1-419_, NP_121-419_, and NP_250-365_ stability was evaluated in the presence of different formulation conditions during storage. In comparison to the original phosphate buffer of 10 mM pH 8.0, 50 mM borax buffer with 0.5 M trehalose was found to be a better option for stabilizing the proteins, Figure 8. However, the borax formulation does not go well in our case for manufacturing the gold nanoparticles (GNPs) of LFIAs and destabilizes the nanoparticles.

## 4. Conclusions

The approach adopted in the work involves reducing the size of a nucleocapsid antigen to enhance its stability. This is conducted while maintaining the required epitopes for antibody identification. This approach can significantly improve diagnostic tests’ accuracy and efficiency. One of the previous studies has shown that the truncation of antigens is the key to improving the sensitivity of nucleocapsid antigens. It demonstrated that the C-terminal domain shows better sensitivity than the N-terminal; however, details of stability or manufacturability were not presented [7]. Integrating theoretical knowledge about B-cell epitope occurrence with peptide stability helped to identify the stretch which was experimentally quite stable. This was probably due to a decrease in net positive charge on the protein. Coincidently, this stable region was also identified as the CTD domain, which interacts with SARS-CoV2 RNA [20].

Stability-enhanced diagnostic antigens are less prone to denaturation or degradation during storage and assay procedures. We have also shown that the stability of the nucleocapsid can be improved by using a suitable formulation buffer (borax buffer with trehalose). However, such a formulation buffer does not support the further downstream process of manufacturing stable gold nanoparticles. We have shown the improved stability of the theoretically identified truncated short nucleocapsid protein over the native protein. We also assume that this will increase the quality of portable testing devices, such as the Lateral Flow Immunoassay (LFIA). Improved stability can ensure the diagnostic test performs consistently and reliably over time due to this critical quality attribute. Smaller antigens with enhanced stability are less likely to interact with unrelated antibodies or molecules, minimizing potential cross-reactivity issues. Specificity is crucial in diagnostic tests to avoid interference and improve test accuracy [21].

This kind of strategy can be used to improve diagnostic antigen stability for new and existing LFIA devices. These devices are intended for use in resource-limited settings and remote areas, improving diagnostic service accessibility. The strategy adopted for the improvement of stability for diagnostic applications can be applied in various fields other than diagnostics. This is particularly true in the contexts of immunology, biotechnology, and medicine.

## Figures and Tables

**Table 1:** Host strains and plasmids used.

Strains/Plasmids	Characteristics	Source
**Strains**		
DH5α	F^+^ endA1 glnV44 thi1 recA1 relA1 gyrA 6 deoR nupG purB20 φ80dlacZΔM15 Δ(lacZYA-argF) U169, hsdR17(r_K_^–^m_K_^+^), λ	Amersham Biosciences, Piscataway, NJ, USA
BL21(DE3)	F^–^ ompT gal dcm lon hsdS_B_(r_B_^–^m_B_^–^) λ (DE3 [lacI lacUV5 T7p07 ind1 sam7 nin5]) [malB^+^]_K-12_(λ^S^)	Novagen, Madison, WI, USA
**Plasmids**		
pET-28b (5369 bp)	Kan, T7 promoter, lacO	GenScript, Piscataway, NJ, USA

**Table 2:** Primer details with the oligonucleotide sequence and the restriction sites inserted. FP: Forward primer and RP: Reverse primer. Restriction sites inserted are marked by underline. Codons for glycine are marked in bold.

S. No.	Plasmid	Oligonucleotide Sequence	Restriction Site
**1.**	pET-28- NP_121-419_	FP-ACCCCATG**GG****A**TCAGCCGCAGAAGCCAG RP-CACGGATCCGGGAAGGTTTTATATGCATCAATATGC	*NcoI* *BamHI*
**2.**	pET-28- NP_250-365_	FP-GTCCCATG**G****GT**CTGCCTTATGGCGCCAATAAG RP-TTCGGATCCGCCTGGGTG	*NcoI* *BamHI*

**Table 3:** Theoretical estimate of the instability index and pI of the proteins with ProtParam analysis.

Theoretical Calculations	NP_1-419_	NP_121-419_	NP_250-365_
**Instability Index**	55.09	52.6	36.28
**pI**	10.07	9.94	9.60

## Data Availability

Data is contained within this article and supplementary material.

## Supplementary Materials

The following supporting information can be downloaded at: https://www.mdpi.com/article/10.3390/biom13101524/s1, Figure S1: Cloning strategies for Nucleocapsid proteins; Figure S2: Expression and partition studies of NP_1-419_, NP_121-419_ and NP_250-365_; Figure S3: Purification studies of NP_1-419_, NP_121-419_ and NP_250-365_; Figure S4: Prptide mapping of purified proteins; Figure S5: Western blot of purified proteins; Figure S6: Stability test of purified proteins.

## References

[1] Li G., Li W., Fang X., Song X., Teng S., Ren Z., Hu D., Zhou S., Wu G., Li K. (2021). Expression and purification of recombinant SARS-CoV-2 nucleocapsid protein in inclusion bodies and its application in serological detection. Protein Expr. Purif..

[2] Zhou Y., Wu Y., Ding L., Huang X., Xiong Y. (2021). Point-of-care COVID-19 diagnostics powered by lateral flow assay. Trends Anal. Chem. TRAC.

[3] Di Nardo F., Chiarello M., Cavalera S., Baggiani C., Anfossi L. (2021). Ten Years of Lateral Flow Immunoassay Technique Applications: Trends, Challenges and Future Perspectives. Sensors.

[4] Mariano G., Farthing R.J., Lale-Farjat S.L.M., Bergeron J.R.C. (2020). Structural Characterization of SARS-CoV-2: Where We Are, and Where We Need to Be. Front. Mol. Biosci..

[5] Okba N.M.A., Müller M.A., Li W., Wang C., GeurtsvanKessel C.H., Corman V.M., Lamers M.M., Sikkema R.S., de Bruin E., Chandler F.D. (2020). Severe Acute Respiratory Syndrome Coronavirus 2-Specific Antibody Responses in Coronavirus Disease Patients. Emerg. Infect. Dis..

[6] Cheng M.P., Papenburg J., Desjardins M., Kanjilal S., Quach C., Libman M., Dittrich S., Yansouni C.P. (2020). Diagnostic Testing for Severe Acute Respiratory Syndrome-Related Coronavirus 2: A Narrative Review. Ann. Intern. Med..

[7] Wu C., Qavi A.J., Hachim A., Kavian N., Cole A.R., Moyle A.B., Wagner N.D., Sweeney-Gibbons J., Rohrs H.W., Gross M.L. (2021). Characterization of SARS-CoV-2 nucleocapsid protein reveals multiple functional consequences of the C-terminal domain. iScience.

[8] Di D., Dileepan M., Ahmed S., Liang Y., Ly H. (2021). Recombinant SARS-CoV-2 Nucleocapsid Protein: Expression, Purification, and Its Biochemical Characterization and Utility in Serological Assay Development to Assess Immunological Responses to SARS-CoV-2 Infection. Pathogens.

[9] Zeng W., Liu G., Ma H., Zhao D., Yang Y., Liu M., Mohammed A., Zhao C., Yang Y., Xie J. (2020). Biochemical characterization of SARS-CoV-2 nucleocapsid protein. Biochem. Biophys. Res. Commun..

[10] Djukic T., Mladenovic M., Stanic-Vucinic D., Radosavljevic J., Smiljanic K., Sabljic L., Devic M., Cujic D., Vasovic T., Simovic A. (2021). Expression, purification and immunological characterization of recombinant nucleocapsid protein fragment from SARS-CoV-2. Virology.

[11] Yue L., Cao H., Xie T., Long R., Li H., Yang T., Yan M., Xie Z. (2021). N-terminally truncated nucleocapsid protein of SARS-CoV-2 as a better serological marker than whole nucleocapsid protein in evaluating the immunogenicity of inactivated SARS-CoV-2. J. Med. Virol..

[12] Khan W.H., Khan N., Mishra A., Gupta S., Bansode V., Mehta D., Bhambure R., Ansari M.A., Das S., Rathore A.S. (2022). Dimerization of SARS-CoV-2 nucleocapsid protein affects sensitivity of ELISA based diagnostics of COVID-19. Int. J. Biol. Macromol..

[13] Omasits U., Ahrens C.H., Müller S., Wollscheid B. (2014). Protter: Interactive protein feature visualization and integration with experimental proteomic data. Bioinformatics.

[14] IEDB Analysis Resource. http://tools.iedb.org/bcell/result/.

[15] Jespersen M.C., Peters B., Nielsen M., Marcatili P. (2017). BepiPred-2.0: Improving sequence-based B-cell epitope prediction using conformational epitopes. Nucleic Acids Res.

[16] Guruprasad K., Reddy B.V., Pandit M.W. (1990). Correlation between stability of a protein and its dipeptide composition: A novel approach for predicting in vivo stability of a protein from its primary sequence. Protein Eng..

[17] Expasy Protparam. https://web.expasy.org/protparam/.

[18] He F. (2011). Laemmli-SDS-PAGE. Bio-Protocol.

[19] He S., Wang J., Chen H., Qian Z., Hu K., Shi B., Wang J. (2023). A Competitive Panning Method Reveals an Anti-SARS-CoV2 Nanobody Specific for an RBD-ACE2 Binding Site. Vaccines.

[20] Yang M., He S.H., Chen X.X., Huang Z.X., Zhou Z.L., Zhou Z.C., Chen Q.Y., Chen S.D., Kang S.S. (2021). Structural Insight Into the SARS-CoV2 Nucleocapsid Protein C-Terminal Domain Reveals a Novel Recognition Mechanism for Viral Transcriptional Regulatory Sequences. Front. Chem..

[21] Virginio V.G., Hernández A., Rott M.B., Monteiro K.M., Zandonai A.F., Nieto A., Zaha A., Ferreira H.B. (2003). A set of recombinant antigens from Echinococcus granulosus with potential for use in the immunodiagnosis of human cystic hydatid disease. Clin. Exp. Immunol..

