# Supplementary material for: Improved Stability and Manufacturability of Nucleocapsid Antigens for SARS-CoV2 Diagnostics through Protein Engineering
Source: Biomolecules. 2023 Oct 14;13(10):1524. doi: 10.3390/biom13101524 (PMC10604654; doi:10.3390/biom13101524)
Supplement: Supplementary file 1 [file biomolecules-13-01524-s001.zip › biomolecules-2601473-supplementary.pdf]

Supplementary File

1. Cloning strategies for nucleocapsid proteins (Sup Figure S1)

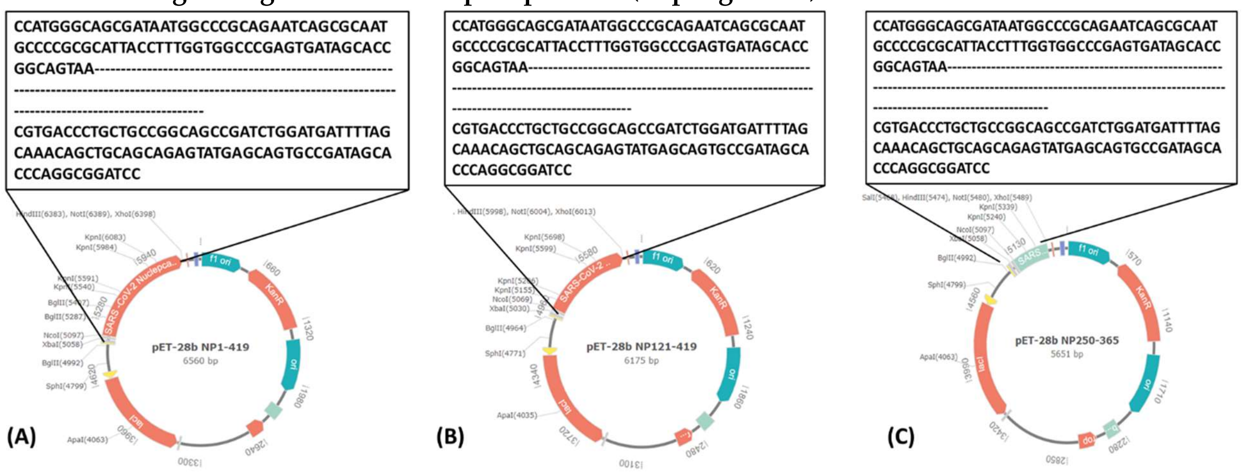

Figure S1. (A): Schematic diagram for SARS-CoV-2 NP_{1-419} cloned in pET-28b vector, **(B):** Schematic diagram for SARS-CoV-2 $NP_{121-419}$ cloned in pET-28b vector and **(C):** Schematic diagram for SARS-CoV-2 $NP_{250-365}$ cloned in pET-28b vector.

2. Expression and partitioning studies of NP_{1-419}, $NP_{121-419}$ and $NP_{250-365}$ (Sup Figure S2)

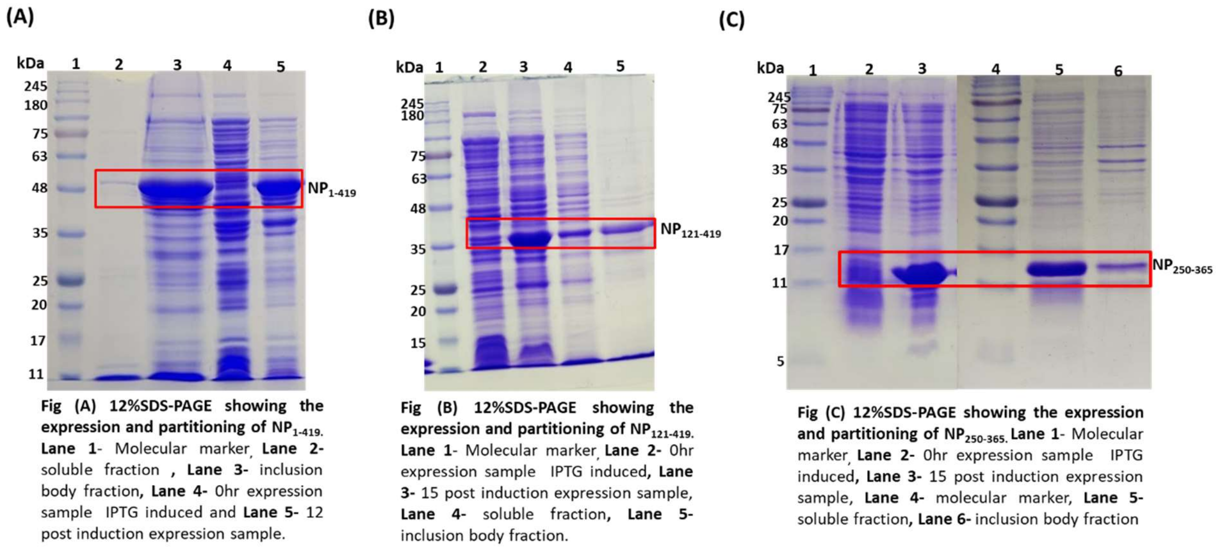

Figure S2. Analysis of Nucleocapsid protein and its variants expression and partitioning studies on 12% tris-glycine SDS-PAGE.

3. Purification studies of NP_{1-419}, $NP_{121-419}$ and $NP_{250-365}$. (Sup Figure S3)

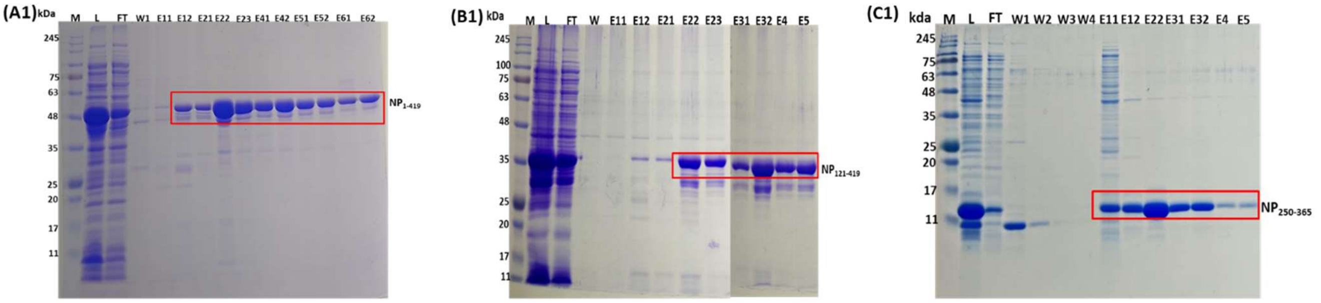

(A2)

Name	Description
M	Marker
L	Load
FT	Flow through
W1	Wash 1% 5mM imidazole
E11	Elution 20% 100mM imidazole
E12	Elution 20% 100mM imidazole (Peak)
E21	Elution 30% 150mM imidazole
E22	Elution 30% 150mM imidazole(Peak)
E23	Elution 30% 150mM imidazole (tail)
E41	Elution 40% 200mM imidazole
E42	Elution 40% 200mM imidazole (Peak)
E51	Elution 50% 250mM imidazole
E52	Elution 50% 250mM imidazole
E61	Elution 100% 500mM imidazole
E62	Elution 100% 500mM imidazole

(B2)

Name	Description
M	Marker
L	Load
FT	Flow through
W1	Wash 10% 50mM imidazole
E11	Elution 20% 100mM imidazole
E12	Elution 20% 100mM imidazole (Peak)
E21	Elution 30% 150mM imidazole
E22	Elution 30% 150mM imidazole(Peak)
E23	Elution 30% 150mM imidazole (tail)
E31	Elution 40% 200mM imidazole
E32	Elution 40% 200mM imidazole (Peak)
E4	Elution 50% 250mM imidazole (Peak)
E5	Elution 100% 250mM imidazole

(C2)

Name	Description
M	Marker
L	Load
FT	Flow through
W1	Wash 1% 5mM imidazole
W2	Wash 2% 10mM imidazole
W3	Wash 5% 25mM imidazole
W4	Wash 10% 50mM imidazole
E11	Elution 20% 100mM imidazole
E12	Elution 20% 100mM imidazole (Peak)
E22	Elution 30% 150mM imidazole(Peak)
E31	Elution 40% 200mM imidazole
E32	Elution 40% 200mM imidazole (Peak)
E4	Elution 50% 250mM imidazole (Peak)
E5	Elution 100% 500mM imidazole

Fig (A1&A2) 12% SDS-PAGE showing the purification of NP_{1-419} and its elution gradient description, respectively. **Fig (B1&B2)** 12% SDS-PAGE showing the purification of $NP_{121-419}$ and its elution gradient description, respectively. **Fig (C1&C2)** 12% SDS-PAGE showing the purification of $NP_{250-365}$ and its elution gradient description, respectively.

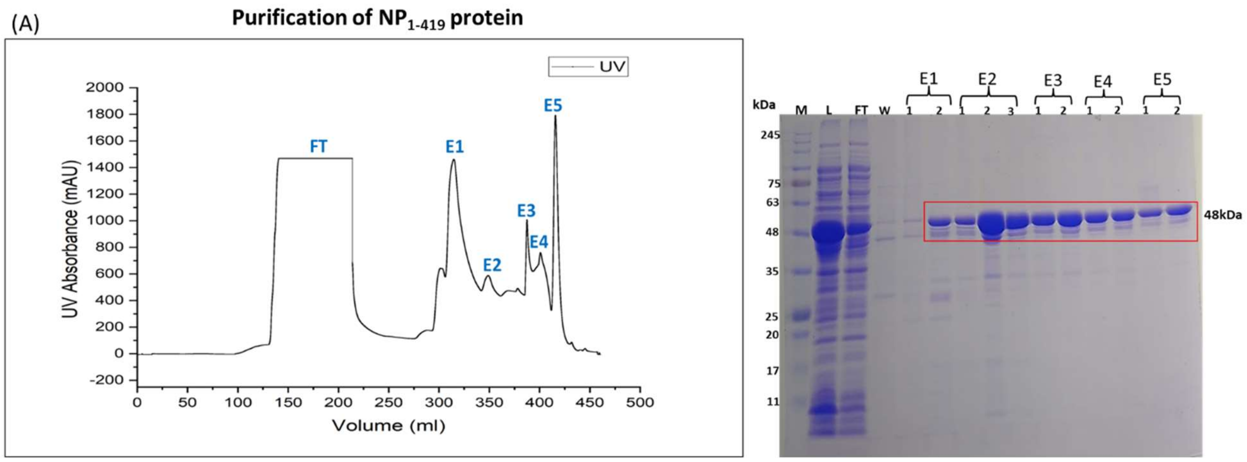

Figure A. Chromatogram and 12% SDS PAGE gel showing purification of NP_{1-419} by Ni-NTA resins. M: Molecular weight marker, L: Protein (Supernatant after lysis) loaded, FT: Flow-through, W1: wash with equilibration buffer,, E1: 1st elution with 50mM Imidazole. E2: 2nd elution with 100mM Imidazole, E3: 3rd elution with 200mM Imidazole, E4: 4th peak with 300 mM Imidazole and E5: 5th peak with 500mM Imidazole

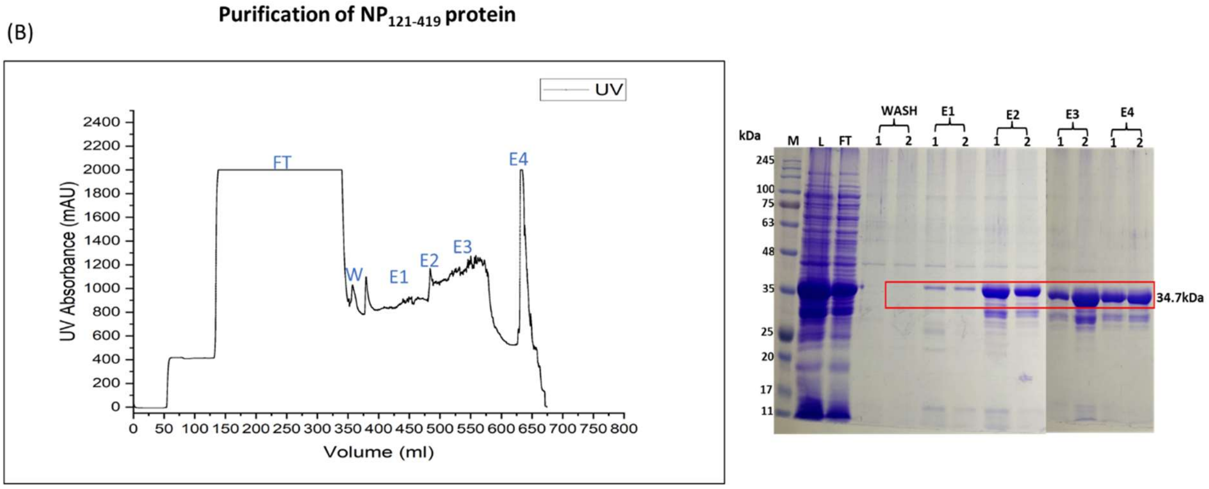

Figure B. Chromatogram and 12% SDS PAGE gel showing purification of NP121-419 by Ni-NTA resins. M: Molecular weight marker, L: Protein (Supernatant after lysis) loaded, FT: Flow-through, W1: wash with equilibration buffer,, E1: 1st elution with 50mM Imidazole. E2: 2nd elution with 100mM Imidazole, E3: 3rd elution with 250mM Imidazole and E4: 4th peak with 500 mM Imidazole.

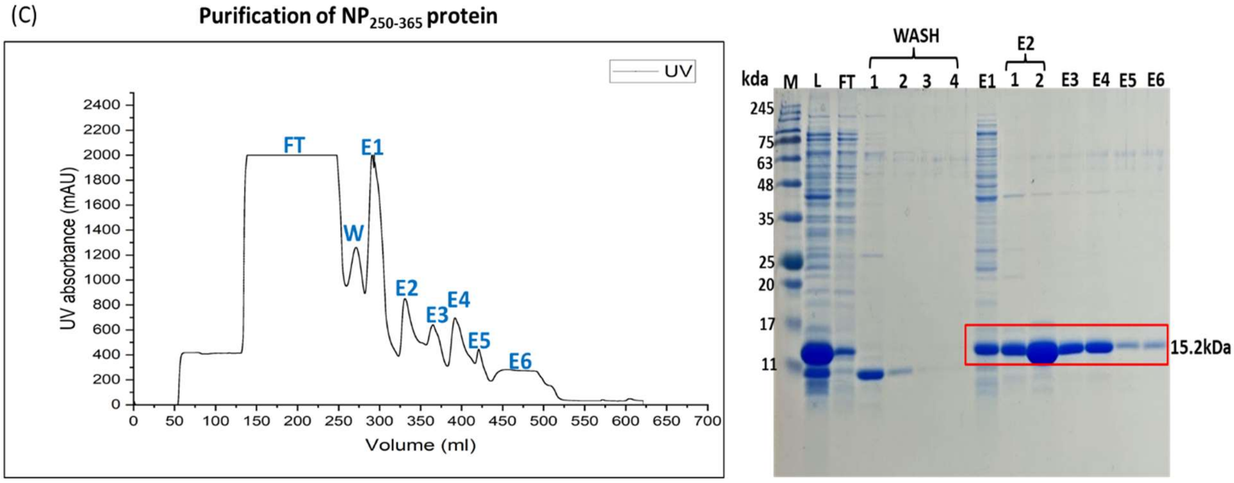

Figure C. Chromatogram and 12% SDS PAGE gel showing purification of NP250-365 by Ni-NTA resins. M: Molecular weight marker, L: Protein (Supernatant after lysis) loaded, FT: Flow-through, W1-W4: wash with equilibration buffer,, E1: 1st elution with 50mM Imidazole. E2: 2nd elution with 100mM Imidazole, E3: 3rd elution with 200mM Imidazole, E4: 4th peak with 300 mM Imidazole, E5: 5th peak with 400mM Imidazole and E6: 6th peak with 500mM Imidazole.

4. Peptide mapping of purified proteins (Sup Figure S4)

(A) Representative sequence coverage map of $NP_{1\text{-}419}$ when compared to *in-silico* sequence.

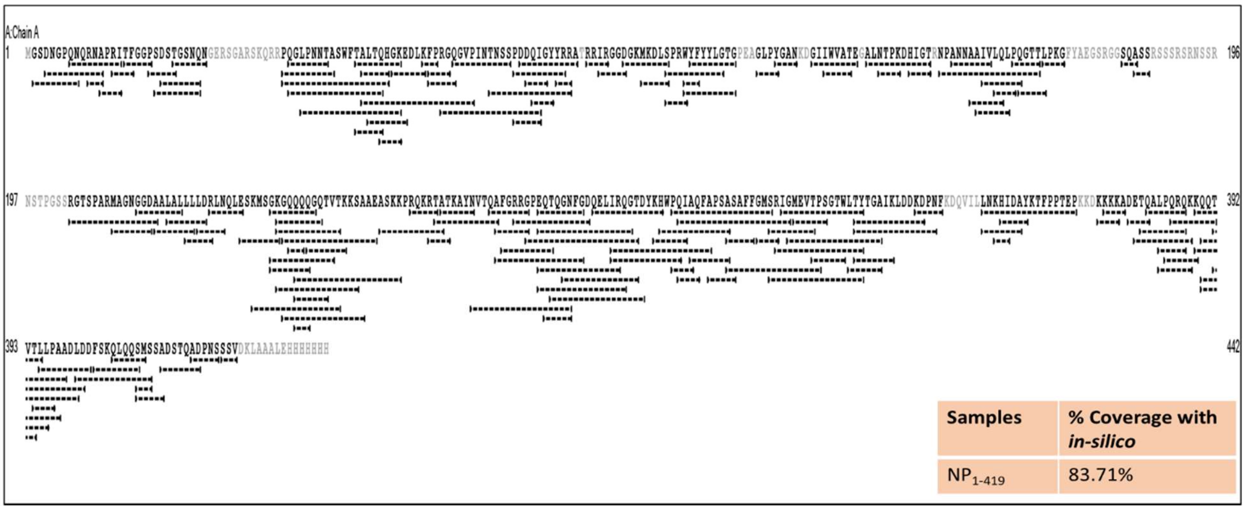

Samples	% Coverage with *in-silico*
$NP_{1\text{-}419}$	83.71%

(B) Representative sequence coverage map of $NP_{121\text{-}419}$ when compared to *in-silico* sequence.

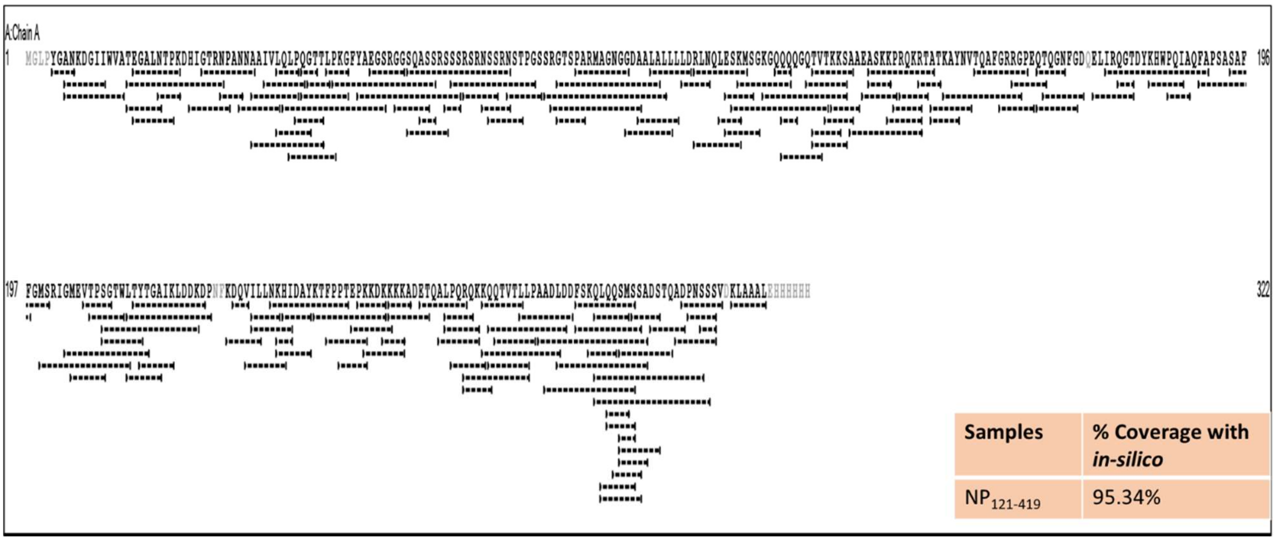

Samples	% Coverage with *in-silico*
$NP_{121\text{-}419}$	95.34%

(C) Representative sequence coverage map of $NP_{250-365}$ when compared to *in-silico* sequence.

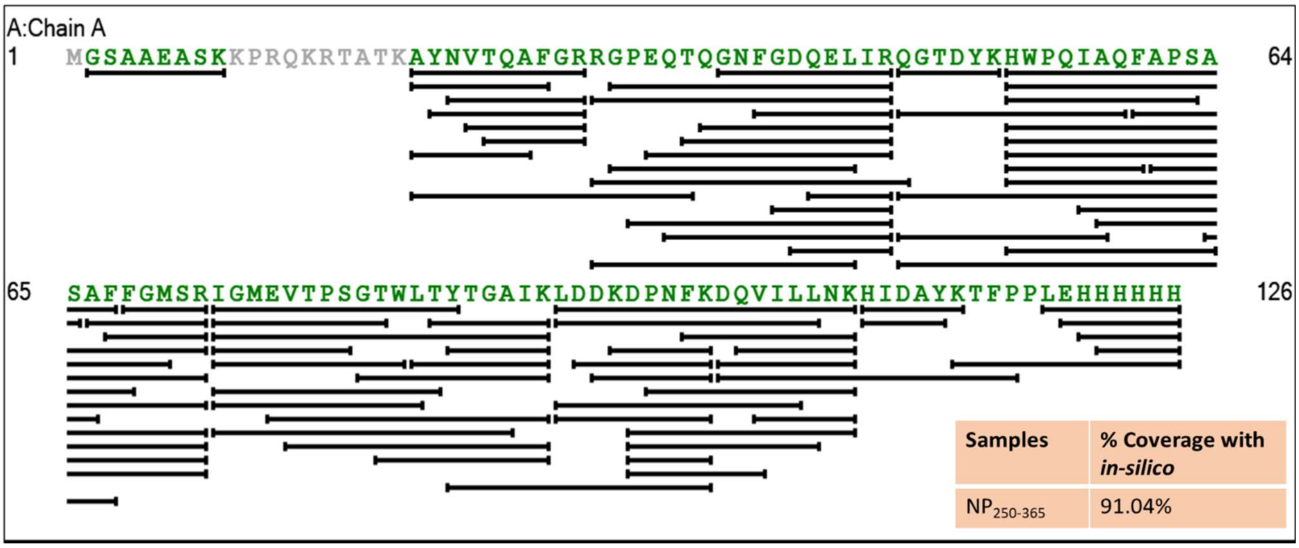

5. Western blot of purified proteins (Sup Figure S5)

WESTERN BLOT ANALYSIS OF NUCLEOCAPSID PROTEINS (NP_{1-419}, $NP_{121-419}$ AND $NP_{250-365}$)

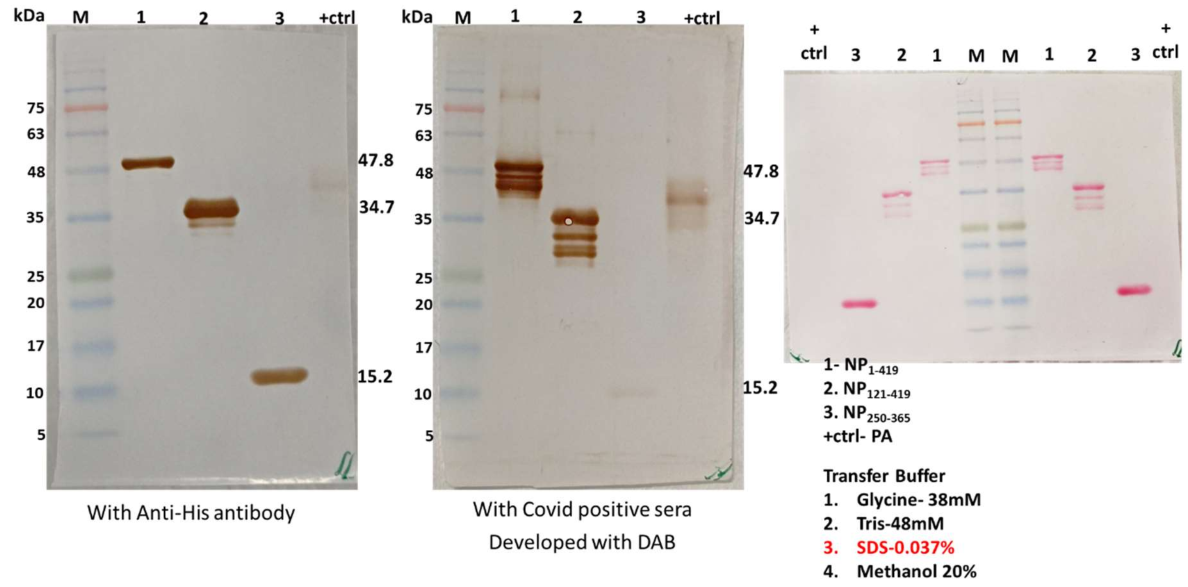

SUP-Figure S5: Ponceau stained NC membrane for NP_{1-419},$NP_{121-419}$ AND $NP_{250-365}$. Western blot is done with anti-His antibody as well as SARS-COV-2 positive human sera

6. Stability test of purified proteins (Sup Figure S6)Analysis by SDS-PAGE

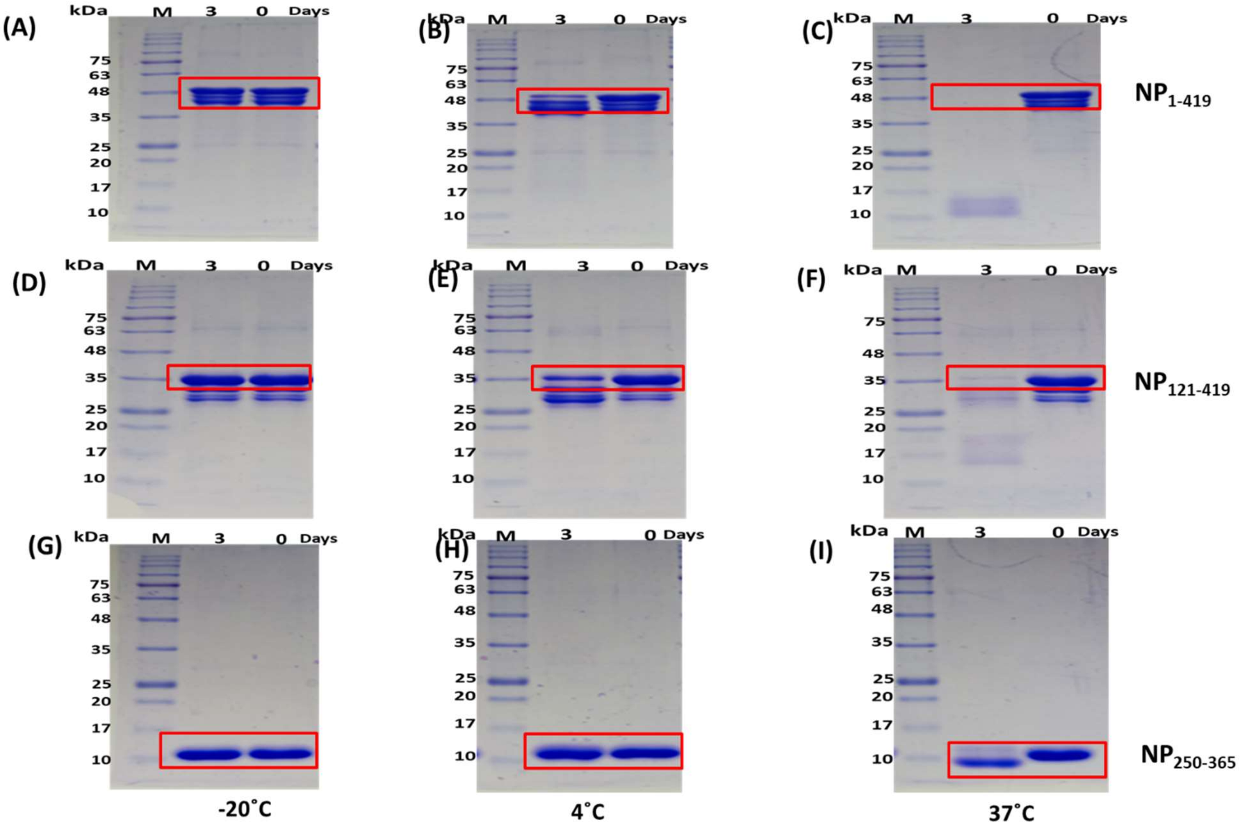

SUP-Figure S6 A: Degradation pattern of NP1-419, NP121-419 and NP250-365 proteins analysed on 12 % SDS-PAGE: In each lane 10 µg of protein charged for stability at different temperatures. NP1-419 Gel (**A-C**): temperature -20 ˚C, 4 ˚C and 37 ˚C, respectively. NP121-419 Gel (D-F) Temperature -20 ˚C, 4 ˚C and 37 ˚C, re-spectively. NP250-365 Gel (G-I) Temperature -20 ˚C, 4 ˚C and 37 ˚C, respectively. In each gel, Lane 1-3: Molecular marker, Day 3, and Day 0 samples, respectively.

B) Analysis by Mass

(I)

NP1-419 -20˚C (3days)

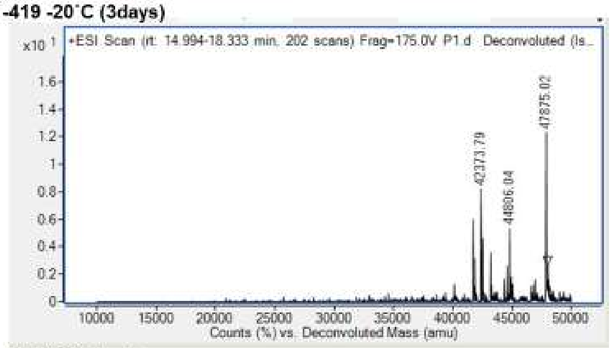

NP1-419 4˚C (3days)

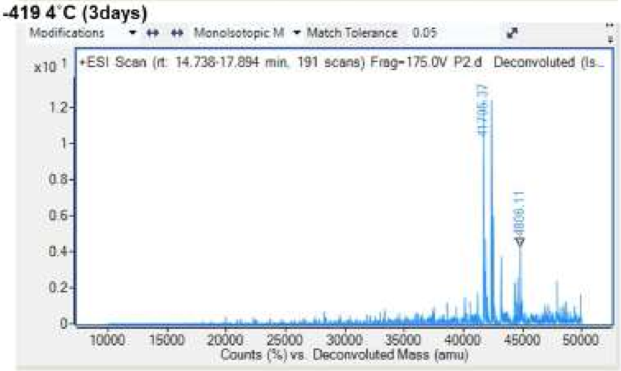

NP1-419 37˚C (3days)

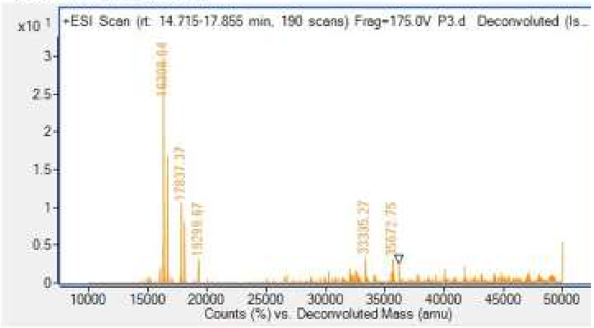

(II)

NP121-419 -20˚C (3days)

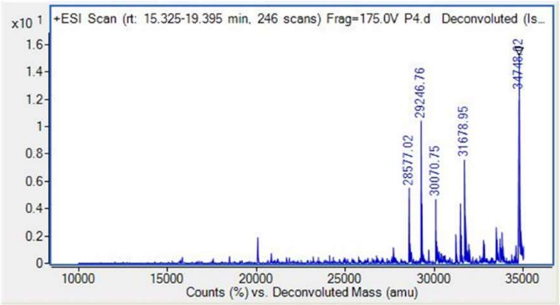

NP121-419 4˚C (3days)

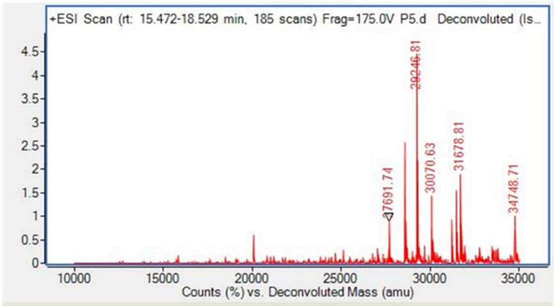

NP121-419 37˚C (3days)

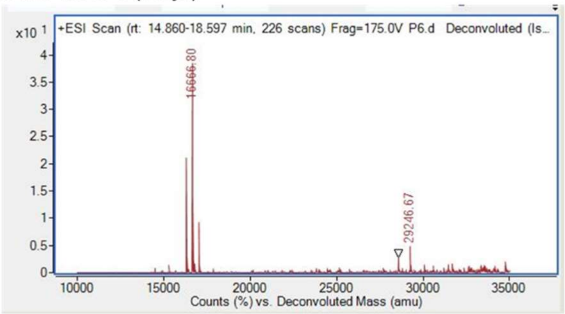

(III)

NP250-365 -20˚C (3days)

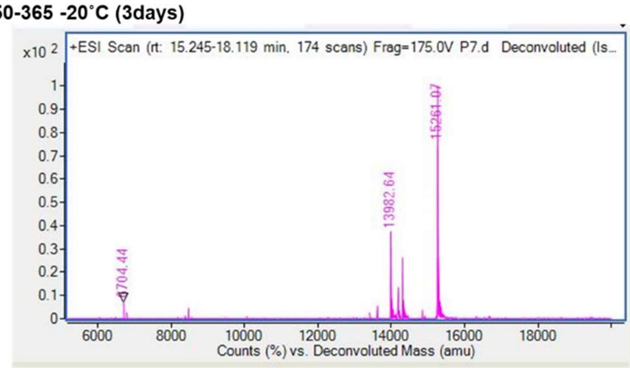

NP250-365 4˚C (3days)

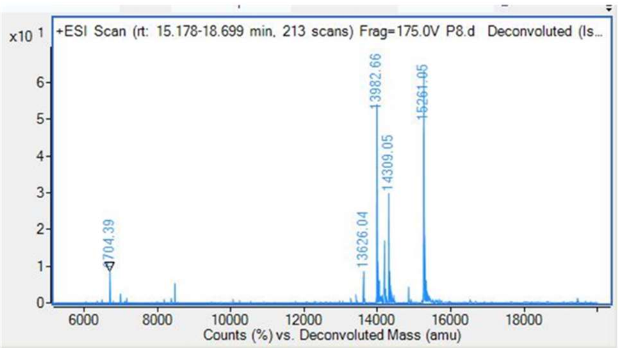

NP250-365 37˚C (3days)

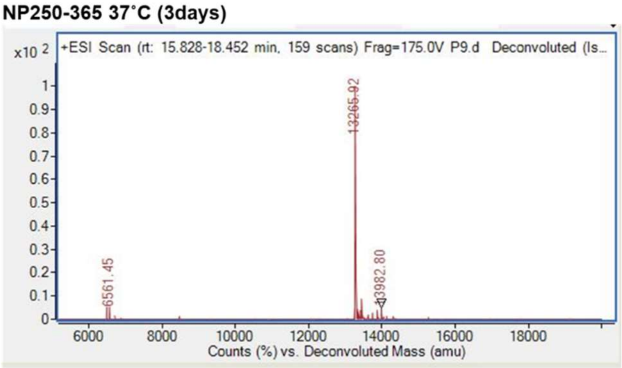

SUP-Figure S6 B: Degradation pattern of NP1-419, NP121-419 and NP250-365 proteins analysed on LCMS: In each 5 µg of protein charged for stability at different temperatures. (I) NP1-419: temperature -20 ˚C, 4 ˚C and 37 ˚C, respectively. (II) NP121-419: Temperature -20 ˚C, 4 ˚C and 37 ˚C, re-spectively. (III) NP250-365 Temperature -20 ˚C, 4 ˚C and 37 ˚C, respectively. In each panel 3rd day, analysis of 3rd sample is presented
